# The impact of voluntary wheel-running exercise on hippocampal neurogenesis and behaviours in response to nicotine cessation in rats

**DOI:** 10.1007/s00213-024-06705-7

**Published:** 2024-10-27

**Authors:** Magdalena Zaniewska, Sabina Brygider, Iwona Majcher-Maślanka, Dawid Gawliński, Urszula Głowacka, Sława Glińska, Łucja Balcerzak

**Affiliations:** 1 Department of Pharmacology and Brain Biostructure, Maj Institute of Pharmacology, Polish Academy of Sciences, 12 Smętna Street, Kraków, 31-343 Poland; 2 Department of Drug Addiction Pharmacology, Maj Institute of Pharmacology, Polish Academy of Sciences, Smętna 12 Street, Kraków, 31-343 Poland; 3 Affective Cognitive Neuroscience Laboratory, Maj Institute of Pharmacology, Polish Academy of Sciences, 12 Smętna Street, Kraków, 31-343 Poland; 4 Faculty of Biology and Environmental Protection, Laboratory of Microscopic Imaging and Specialized Biological Techniques, University of Lodz, Banacha 12/16, Lodz, 90-237 Poland; 5 Present Address: Department of Physiology, Jagiellonian University Medical College, 16 Grzegorzecka Street, Kraków, 31- 531 Poland

**Keywords:** Doublecortin, Hippocampal neurogenesis, Nicotine self-administration, Rat, Wheel running

## Abstract

**Rationale:**

The literature indicates that nicotine exposure or its discontinuation impair adult hippocampal neurogenesis in rats, though the impact of exercise on this process remains unclear. We have previously shown that disturbances in the number of doublecortin (DCX, a marker of immature neurons)-positive (DCX^+^) cells in the dentate gyrus (DG) of the hippocampus during nicotine deprivation may contribute to a depression-like state in rats.

**Objectives:**

This study aimed to investigate the effect of running on hippocampal neurogenesis, depression-like symptoms, and drug-seeking behaviour during nicotine deprivation.

**Methods:**

The rats were subjected to nicotine (0.03 mg/kg/inf) self-administration via an increasing schedule of reinforcement. After 21 sessions, the animals entered a 14-day abstinence phase during which they were housed in either standard home cages without wheels, cages equipped with running wheels, or cages with locked wheels.

**Results:**

Wheel running increased the number of *K*_i_-67^+^ and DCX^+^ cells in the DG of both nicotine-deprived and nicotine-naive rats. Wheel-running exercise evoked an antidepressant effect on abstinence Day 14 but had no effect on nicotine-seeking behaviour on abstinence Day 15 compared to rats with locked-wheel access.

**Conclusions:**

In summary, long-term wheel running positively affected the number of immature neurons in the hippocampus, which corresponded with an antidepressant response in nicotine-weaned rats. One possible mechanism underlying the positive effect of running on the affective state during nicotine cessation may be the reduction in deficits in DCX^+^ cells in the hippocampus.

**Supplementary Information:**

The online version contains supplementary material available at 10.1007/s00213-024-06705-7.

## Introduction

Nicotine, the principal component in tobacco products, such as cigarettes, cigars, and smokeless cigarette products, including heat-not-burn devices, is implicated in tobacco addiction in humans. The development of nicotine dependence is linked to the binding of nicotine to nicotinic acetylcholine receptors (nAChR), especially the α_4_β_2_ receptor subtypes, in the brain (Mineur and Picciotto 2008).

Nicotine dependence poses a severe public health concern, with estimates indicating that more than 1 billion people worldwide are regular smokers (The World Health Organization (WHO) 2021). Smoking cessation leads to severe withdrawal symptoms, including somatic signs such as bradycardia, hunger, fatigue, headache, and gastrointestinal problems, as well as affective symptoms such as increased craving, irritability, anxiety, and depression (Crocq 2003; Zaniewska et al. 2009). Several therapies have been developed to help individuals quit smoking. The most effective treatments include nicotine e-cigarettes or medicines designed to either replace nicotine (combination nicotine replacement therapy) or partially mimic its effects (partial nAChR agonists) (Lindson et al. 2023). Despite these efforts, an ongoing controversy regarding the use of pharmacological interventions in specific populations, such as adolescents, has resulted in the absence of treatments approved by the Food and Drug Administration (FDA) for these individuals (Kaplan and Ivanov 2011). In such cases, non-pharmacological treatment options, such as psychosocial interventions, take precedence in managing tobacco addiction.

Emerging evidence indicates that exercise has potential as a non-pharmacological method for reducing nicotine craving and alleviating withdrawal symptoms in both young and adult smokers. Clinical research has demonstrated that short bouts of exercise during acute abstinence improve quitting intentions and reduce craving (Ussher et al. 2001; Janse et al. 2009; Elibero et al. 2011; De Jesus and Prapavessis 2018; Georgakouli et al. 2021). During initial abstinence, moderate-intensity exercise was shown to be more effective for alleviating withdrawal symptoms in young adults than vigorous-intensity exercise (Everson et al. 2008). Short bouts of moderate and vigorous exercise during the 6-week abstinence period ameliorated psychological withdrawal symptoms without affecting smoking abstinence (Ussher et al. 2003). Leisure-time, moderate, or vigorous exercise in a 12-week smoking cessation program coincided with relief of withdrawal symptoms, craving and/or increased smoking cessation (Bock et al. 1999; Marcus et al. 1999; Harrison et al. 2019; Kunicki et al. 2022). Preclinical studies conducted in an adolescent-onset model have indicated that physical activity may prevent vulnerability to nicotine self-administration in rats (Sanchez et al. 2015). When adolescent rats were given access to running wheels during nicotine abstinence, they exhibited decreased responses during the extinction sessions (Sanchez et al. 2013, 2014; Lynch et al. 2019), and only in adolescent females, did running decrease cue-induced reinstatement (Lynch et al. 2019; Sanchez et al. 2013). To date, data on the effects of running on nicotine-seeking behaviour in adult rats are lacking in the literature. Moreover, the impact of exercise on the emotional aspects of rats deprived of self-administered nicotine has not yet been examined.

Voluntary wheel running serves as an animal model for studying the beneficial effects of aerobic exercise training observed in humans (Mul 2018). It has been shown to enhance various physiological aspects, such as cerebral blood flow and angiogenesis, and maintain the integrity of the blood‒brain barrier (Vecchio et al. 2018). Research suggests that exercise can help individuals manage withdrawal symptoms by increasing the release of β-endorphins in the brain, acting as a reinforcer similar to that of smoking (Ernst et al. 2006; Mul 2018; Georgakouli et al. 2021). In rodents, voluntary wheel running has been found to enhance hippocampal neurogenesis (Inoue et al. 2015; Van Praag et al., 1999). This process encompasses several stages, including proliferation (intensive cell divisions), migration, differentiation, maturation, and the functional integration of newly formed neurons into the neural network (Kuhn et al. 1996). Neural progenitor cells (NPCs), which are responsible for the formation of new neurons, have been detected in the subgranular zone (SGZ) of the dentate gyrus (DG) of the hippocampus in rodents, primates and humans (Kuhn et al. 1996; Gould et al. 1998; Eriksson et al. 1998). After several rounds of division, newborn cells migrate to the granule cell layer (GCL) of the DG. NPCs develop into immature neurons that express doublecortin (DCX) and PSA-NCAM (polysialic acid-neural cell adhesion molecule) (Kuhn et al. 1996). Subsequently, the cells differentiate into mature neurons, accompanied by the expression of the neuronal marker NeuN (Kuhn et al. 1996).

Hippocampal neurogenesis is essential for mood regulation and the response to antidepressants (Snyder et al. 2011; Castrén and Hen 2013). It is a vital mechanism that enables adaptation to environmental changes, facilitating learning and managing stress (Thuret et al. 2009; Snyder et al. 2011). In addition to wheel running, factors such as antidepressants or an enriched environment have been shown to increase hippocampal neurogenesis (Malberg et al. 2000; Van Praag et al. 2002), while chronic stress or drugs of abuse, including nicotine, decrease this process, although not in every model (e.g., Abrous et al. 2002; Ahdoot-Levi et al. 2021; Cohen et al. 2015; Deschaux et al. 2014; Lee et al. 2006; Noonan et al. 2008; Wei et al. 2012).

We previously demonstrated that animals deprived of nicotine self-administration for two weeks exhibited a decreased number of surviving (5-bromo-2’-deoxyuridine; BrdU^+^) and immature (DCX^+^) neurons in the DG of the hippocampus (Zaniewska et al. 2021). At the behavioural level, prolonged abstinence from nicotine was associated with an increase in immobility time in the forced swim test (FST), indicating depression-like behaviour, as well as nicotine-seeking behaviour and deficits in cognitive-like behaviour (Zaniewska et al. 2021). Chronic treatment with the serotonin (5-HT)_2C_ receptor agonist lorcaserin improved depression-like behaviour and concurrently restored the diminished number of DCX^+^ cells. This suggested that the emotional aspects of nicotine cessation could be linked to neuroadaptations in hippocampal neurogenesis, particularly during the neuronal maturation stage.

The objective of the present study was to investigate the effects of chronic wheel running on hippocampal neurogenesis during nicotine abstinence. To model nicotine dependence, we used intravenous nicotine self-administration. During the nicotine deprivation period, the animals were assigned to different housing conditions: standard home cages (control group) or larger cages with wheels either locked (enriched environment) or unlocked (enriched environment + wheel running). Our objective was to examine the separate influences of running and environmental enrichment on neurogenesis, particularly during the extended phase of drug cessation. Initially, we examined the influence of long-term running during nicotine cessation on hippocampal neurogenesis. By administering BrdU during the early phase of nicotine cessation, we were able to label proliferating cells in the DG of the hippocampus and track their fate (survival) within the extended drug cessation microenvironment. Subsequently, in another cohort of animals, on Days 14 and 15 of abstinence, we compared the behaviour in the FST, locomotor response, and strength of nicotine seeking between rats exposed to wheel-running exercise and those with access to locked wheels throughout the drug cessation period.

## Materials and methods

### Animals

Experiments were conducted on adult male Sprague‒Dawley rats (*n* = 50; 186–237 g) acquired from a licenced animal breeder (Charles River, Sulzfeld, Germany). Before surgery, a total of 4–5 animals were housed per cage (59 cm × 38 cm × 20 cm), or after surgery, they were housed individually in standard cages (42 cm × 26.5 cm × 18 cm) in a colony room (temperature: 22 ± 2 °C; humidity: 55 ± 10%; 12-h light-dark cycle; lights on at 6:00 h). The animal’s condition was monitored once or twice (after the surgery) daily by the experimenter to ensure their welfare and to detect any signs of distress or illness. The animals had free access to rodent chow throughout the entire experiment. Water was also available ad libitum, except for during the initial training sessions. All experiments were conducted during the early light phase (between 7:00 AM and 2:00 PM) of the light‒dark cycle. The experiments were performed in compliance with the European Community Council Directive 2010/63/EU for Animal Experiments, and were approved by the II Local Ethics Committee at the Maj Institute of Pharmacology, Polish Academy of Sciences (permission numbers: 1240/2015, 1273/2015, 186/2016, 161/2017, 353/2017, 354/2017).

### Drugs

The following drugs were used: a thymidine analogue that labels proliferating cells, BrdU (Sigma-Aldrich, USA), and (-)-nicotine hydrogen tartrate salt (Sigma-Aldrich, St. Louis, MO, USA; cat #N5260–25G). The preparation and doses of nicotine (expressed as the free base) used were previously reported (Zaniewska et al. 2010; Higgins et al. 2012; Le Foll et al. 2012; Cousins et al. 2014; Cohen et al. 2015; Briggs et al. 2016; Fletcher et al. 2019). BrdU was dissolved in a filtered (Whatman filters, PP w/GMF 0.2 μm; GE Healthcare, USA) solution of 0.9% saline and 0.007 N NaOH (pH 7.5). The BrdU solution was slowly stirred on a heated magnetic stirrer until dissolved. It was then loaded into a syringe and injected intraperitoneally (ip) at a volume of 2 ml/kg when the syringe was comfortably warm to the touch. Nicotine was dissolved in sterile 0.9% saline, and the pH was adjusted to 7.0 using 20% NaOH. The nicotine solution was stored in the dark at 4 °C, but prior to the experiment, it was allowed to warm to room temperature. For intraveous (iv) self-administration, the nicotine solution (0.03 mg/kg/inf) was filtered through Whatman filters. To induce drug-seeking behaviour in the animals, nicotine (0.4 mg/kg) was administered via subcutaneous injection (sc) at a volume of 1 ml/kg.

### The effects of voluntary wheel running on hippocampal neurogenesis during nicotine cessation

Training and intravenous catheter implantation.

**Fig. 1:**
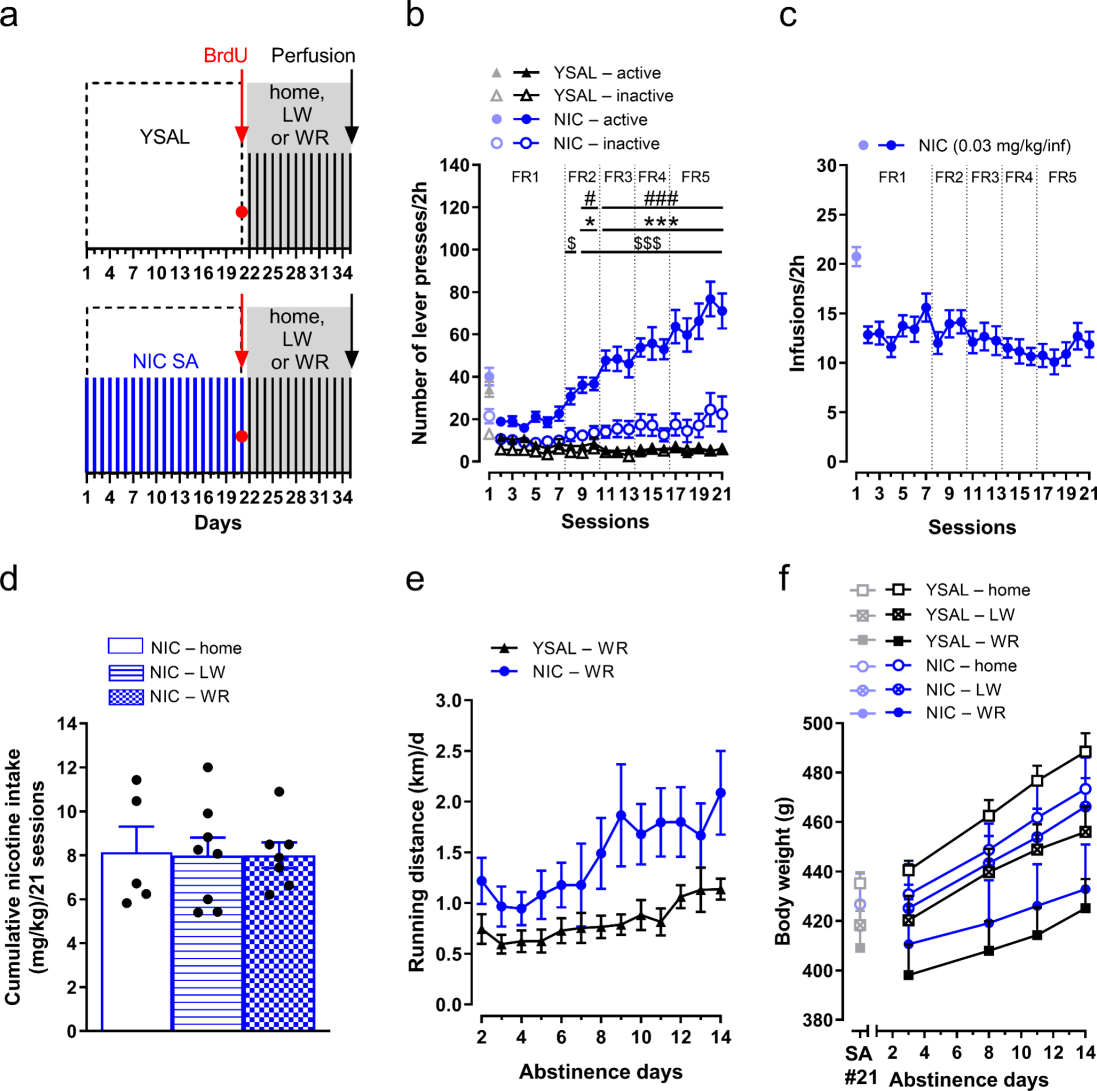
Effects of wheel-running exercise on hippocampal neurogenesis during long-term nicotine deprivation. Rats were allowed to self-administer nicotine (0.03 mg/kg/inf, NIC SA) or received saline infusions (YSAL) in 2-h sessions for 21 days. Immediately after the last self-administration session, the rats were injected with 5-bromo-2’-deoxyuridine (BrdU, 3 × 50 mg/kg, ip) to label proliferating cells. The rats then entered the deprivation phase for 14 days. On abstinence Day 1, the animals were transferred to cages equipped with running wheels (WR) or locked wheels (LW) for the next 13 abstinence days. The control groups remained in their home cages throughout the entire deprivation period (home). On Day 14 of abstinence, the animals were perfused, and hippocampal neurogenesis was examined. (**a**) The experimental schedule for NIC SA and WR exposure during nicotine cessation, followed by neurogenesis analyses. (**b**) The number of lever presses (made under the fixed ratio (FR) schedule, during infusions, and the time-out period) in rats self-administering NIC (*n* = 20) or receiving YSAL (*n* = 18) during an increasing schedule of reinforcement (FR(1–5)). The lever presses in session 1 are shown; however, they were not included in the analysis due to the effect of previous water training on lever pressing on this day. (**c**) NIC infusions in rats (*n* = 20) throughout 21 self-administration sessions. The infusions in session 1 were not included in the analysis due to the effect of previous water training on infusions on this day. (**d**) Cumulative NIC intake (mg/kg) during 21 sessions in the three assigned groups (kept in home cages: *n* = 5; exposed to LW: *n* = 8; exposed to WR: *n* = 7, during nicotine cessation). (**e**) Daily running distance ((km)/d) in the NIC-deprived group (*n* = 7) and YSAL-treated group (*n* = 7) with access to WR. (**f**) Effects of different environmental conditions (home, LW, and WR) on the body weight (**g**) of rats deprived of NIC (*n* = 4–8 rats/group). For comparison, the body weights of rats on the last day of self-administration are shown. The data are expressed as the means (± SEM). (**b**) Post hoc Tukey test: ^*^p < 0.05, ^***^p < 0.001 vs. NIC–inactive lever presses; ^#^*p* < 0.05, ^###^*p* < 0.001 vs. NIC–active lever presses on session 2; ^$^*p* < 0.05, ^$$$^*p* < 0.001 vs. YSAL–active lever presses. (**e**) *p* < 0.05: the effect of NIC deprivation vs. YSAL; *p* < 0.001: the effect of abstinence day: post hoc Tukey: *p* < 0.01 Day 14 vs. Day 2. (**f**) *p* < 0.01: the effect of environment: post hoc Tukey: *p* < 0.01 WR vs. home; *p* < 0.001: the effect of abstinence day: *p* < 0.001 Day 8 vs. 3, Day 11 vs. 3 and 8, Day 14 vs. 3, 8 and 11

Using a previously published protocol (Filip et al. 2007) with small modifications, animals (*n* = 38; Fig. 1a) were trained to press a lever for 2 h in standard operant chambers (Med-Associates, St. Albans, GA, USA) under a fixed ratio (FR) 1 schedule of water reinforcement for 5 days. Prior to training, the animals underwent 16–18 h of water deprivation. After training, the rats were given free access to food and water, and after 2 days, they underwent surgery. Animals were anaesthetized with a solution containing ketamine (20 mg/kg, intramuscular injection (im); Biowet, Poland) and dexmedetomidine (0.1 mg/kg, im; Orion Corporation, Finland) and were then implanted with a silastic catheter in the external right jugular vein, as previously described (Filip et al. 2007). Following catheter implantation, all animals were allowed to recover for 7–10 days. Animals received 4–5 ml of a 0.9% NaCl/5% glucose solution (sc) for two days after surgery and the anti-inflammatory/analgesic drug meloxicam (0.04 mg/kg, sc; Metacam, Boehringer Ingelheim, Ingelheim/Rhein, Germany) for three days. The catheters were flushed daily with 0.2 ml of sterile 0.9% saline solution containing cefazolin (100 mg/ml; Polpharma, Poland), a broad-spectrum antibiotic, and an anticoagulant heparin (100 IU/ml; Polfa, Poland) to maintain catheter function.

#### Maintenance of self-administration

After recovery from surgery, the animals were trained for one day to press a lever under an FR1 schedule of water reinforcement during a 2-h session. Subsequently, the rats were trained to self-administer nicotine (0.03 mg/kg/inf) using an increasing FR (1–5) schedule of reinforcement (Le Foll et al. 2012). During the maintenance phase, rats were given access to nicotine during 2-h daily sessions (6 days/week; 21 sessions). In the initial sessions, animals had limited access to water for 2 h per day immediately after the session (with free access from Saturday after the session until late Sunday afternoon). Over the subsequent days, water access was gradually increased to ad libitum during the second week of self-administration. The nicotine dose given to each individual rat was adjusted by weighing the animals every second or third day to account for their growth. The ambient light was on throughout each session. Each completion of the FR schedule on the active lever resulted in an infusion of nicotine (0.03 mg/kg per 0.1 ml) and a 5-second presentation of a conditioned stimulus (illumination of a stimulus light + tone). After every infusion, there was a 20-s time-out period during which responses were recorded but not reinforced. Rats were tested simultaneously in two groups, with one group serving as the ‘yoked’ control, which received an injection of saline (YSAL) each time a response-contingent injection of nicotine was self-administered by the paired rat from the second group (NIC). Saline passive injections were accompanied by the presentation of cues (light + tone). Acquisition of the conditioned operant response lasted at least 21 days until the subjects achieved stable self-administration over the last three sessions, with a standard deviation within those days that was < 10% of the average. The results are presented as the number of intravenous infusions per session and the number of lever presses, reported separately for the active and inactive levers. The number of lever presses corresponds to all presses performed under the FR schedules, during infusions, and/or time-out periods.

#### Nicotine deprivation

After the last self-administration session, the rats were administered three injections of BrdU (50 mg/kg, ip) at 6-h intervals to label proliferating cells during the early deprivation period. The first BrdU injection was administered 15–20 min after the end of the self-administration session, allowing time to remove the animals from the experimental cages and prepare warm BrdU solution. Twenty-four hours after the last self-administration session, the rats were transferred to large polycarbonate cages (41 cm × 51 cm × 21 cm) equipped with either running wheels (diameter: 35 cm; Campden Instruments Ltd., UK; YSAL– WR: *n* = 7; NIC– WR: *n* = 7) or locked wheels (YSAL– LW: *n* = 7; NIC– LW: *n* = 8) until Day 14 of nicotine abstinence. Animals in the locked wheel condition had access to the wheel but were unable to run, as the wheel was immobilized. The control groups remained in their standard home cages throughout the entire deprivation period (YSAL– HOME: *n* = 4; NIC– HOME: *n* = 5).

In the running conditions, a counter attached to the wheel counted the flag number (6 flags per revolution) during the daily 24-h sessions. The total distance run (in km) for the interval experiment was calculated as the revolution count multiplied by 1.1 m (the distance per revolution) divided by 1000.

#### Immunohistochemistry

At 14 days after cessation, the animals were anaesthetized with a sublethal dose of sodium pentobarbital (90 mg/kg) and pentobarbital (18 mg/kg) (ip; Morbital, PGF Cefarm, Poland) and transcardially perfused with 0.9% NaCl, followed by buffered 4% paraformaldehyde (VWR International, USA). The brains were then isolated and postfixed in a buffered solution of 4% paraformaldehyde for 24 h at 4 °C. After the postfixation period, the brains were cut into 50 μm thick coronal sections at the level of the hippocampus (Bregma = -2.04 to -6.60 mm) according to a stereotaxic atlas of the rat brain (Paxinos and Franklin 2001) using a Leica VT-1000 S vibratome (Leica Microsystems, Heidelberg, Germany). For immunohistochemistry, every ninth section throughout the entire hippocampus was preserved for further processing (7–8 sections from each subject). For immunofluorescence, two sections containing the proximal (Bregma = -2.92 mm) and distal (Bregma = -5.16 mm) parts of the hippocampus were selected. Two methods were used to study neurogenesis: immunoenzymatic labelling and triple immunofluorescence labelling. Immunoenzymatic labelling was used to assess the total number of *K*_i_-67 (marker of cell proliferation)-, BrdU (marker of survival of proliferating cells)-, DCX-, and NeuN-positive cells (*K*_i_-67^+^, BrdU^+^, DCX^+^ or NeuN^+^, respectively). Triple immunofluorescence labelling was performed to determine changes in the differentiation of the surviving cells. The detailed immunostaining protocols used are described in previous publications (Maćkowiak et al. 2005, 2009; Chocyk et al. 2015; Majcher-Maślanka et al. 2019; Zaniewska et al. 2021; Solarz-Andrzejewska et al. 2023).

#### Single staining for BrdU

Survival of dividing progenitor cells was assessed by staining for BrdU two weeks after BrdU injections. Free-floating sections were washed with 0.01 M PBS (pH 7.4) and denatured in a solution containing 50% formamide/2x SSC (saline-sodium citrate buffer, pH 7.0; Sigma-Aldrich) for 2 h at 65 °C. The sections were then washed twice in 2x SSC buffer for 5 min and incubated in 2 M HCl for 30 min at 37 °C before being incubated in 0.1 M borate buffer (0.1 M boric acid + NaOH; pH 8.5) for 10 min at room temperature. Subsequently, the brain sections were rinsed with 0.01 M PBS and incubated in blocking buffer (5% normal goat serum; Vector Laboratories, Peterborough, UK and 0.3% Triton X-100 in 0.01 M PBS) for 1 h. Finally, the sections were incubated (48 h at 4 °C) with primary monoclonal anti-BrdU mouse antibodies (1:200; Roche Diagnostics, Indianapolis, IN, USA; cat #11170376001) in 3% normal goat serum supplemented with 0.3% Triton X-100 in 0.01 M PBS. Reactions were visualized using biotinylated goat anti-mouse IgG (1:200, 1 h; Vector Laboratories), followed by incubation with avidin-biotin-horseradish peroxidase complex (1:200, 1 h; Vectastain Elite ABC Kit, Vector Laboratories), 3,3′-diaminobenzidine tetrahydrochloride (DAB)-nickel solution (0.02% DAB + 0.03% NiCl_2_ in 0.01 M PBS), and 0.01% H_2_O_2_. This resulted in the immunoreactive cells appearing as a dark grey colour. Finally, the sections were rinsed in 0.01 M PBS, mounted on SuperFrost Plus slides (Menzel-Gläser, Thermo Scientific, Braunschweig, Germany), air-dried, and coverslipped using Permount (Fisher Scientific) as the mounting medium.

#### Single staining for *K*_i_-67 or DCX

Free-floating brain sections were washed with 0.01 M PBS (pH 7.4) and blocked in blocking buffer containing 5% normal goat serum for *K*_i_-67 or 5% normal rabbit serum for DCX labelling (Vector Laboratories) and 0.3% Triton X-100 in 0.01 M PBS for 1 h. Then, the sections were incubated with either rabbit polyclonal anti-*K*_i_-67 (1:750; Abcam, Cambridge, UK; cat #ab15580) or goat polyclonal anti-DCX (1:500; Santa Cruz Biotechnology, Santa Cruz, CA, USA; cat #sc-8066) primary antibodies diluted in the appropriate 3% normal serum with 0.3% Triton X-100 in 0.01 M PBS for 48 h at 4 °C. The reaction was visualized using a biotinylated goat anti-rabbit (1:200; Vector Laboratories) or rabbit anti-goat (1:200; Vector Laboratories) IgG and peroxidase complex (1:200, 1 h; Vectastain Elite ABC Kit), followed by incubation with a 0.02% DAB solution and 0.01% H_2_O_2_. For DCX, the immunoreactive cells appeared brown in colour. For *K*_i_-67, a DAB-nickel solution (0.02% DAB + 0.03% NiCl_2_ in 0.01 M PBS) with 0.01% H_2_O_2_ was used, resulting in immunoreactive cells with a dark grey colour. Finally, the sections were rinsed in 0.01 M PBS, mounted on SuperFrost Plus slides (Menzel-Gläser), air-dried, and coverslipped using Permount.

#### Single staining for NeuN

Free-floating sections were washed in 0.01 M PBS (pH 7.4) and incubated in PBS containing 0.3% H_2_O_2_ and 0.2% Triton X-100 for 30 min to block endogenous peroxidase activity. Next, the sections were rinsed and blocked in a solution containing 5% normal goat serum and 0.2% Triton X-100 in PBS for 1 h. After blocking, the sections were incubated with a mouse anti-NeuN antibody (1:1000; Millipore) for 48 h at 4 °C. The antibody was diluted in a solution containing 3% normal goat serum and 0.2% Triton X-100 in PBS. The sections were then washed in PBS and incubated with biotinylated goat anti-mouse IgG (1:200; Vector Laboratories) for 1 h, followed by incubation with an avidin-biotin-peroxidase complex (1:100, 1 h; Vectastain Elite ABC Kit) for 1 h. The immunohistochemical reaction was developed using a solution of 0.02% DAB and 0.01% H_2_O_2_ in TBS, resulting in brown staining of the immunoreactive cells. Finally, the sections were rinsed in 0.01 M PBS, mounted on SuperFrost Plus slides (Menzel-Gläser), air-dried, and coverslipped using Permount.

#### Immunofluorescence staining

The differentiation of BrdU^+^ cells was assessed two weeks after BrdU administration by immunofluorescent triple labelling for BrdU, DCX and NeuN. The sections were first treated to denature DNA (as described above) and then blocked with a buffer containing 5% normal donkey serum (Jackson Immunoresearch Laboratories, West Grove, PA, USA) and 0.3% Triton X-100 in 0.01 M PBS for 1 h. Next, a cocktail of primary antibodies against BrdU (monoclonal rat 1:300; Accurate Chemical and Scientific Corp., Westbury, NY, USA; cat #AB6326), DCX (polyclonal goat 1:500; Santa Cruz Biotechnology) and NeuN (monoclonal mouse 1:1000; EMD Millipore, Temecula, CA, USA; cat #MAB377) in 3% normal donkey serum with 0.3% Triton X-100 in 0.01 M PBS was applied to the sections, which were then allowed to incubate for 48 h at 4 °C. After washing, a mixture of secondary antibodies (Alexa 488-conjugated donkey anti-rat IgG 1:200; Thermo Fisher Scientific, Rockford, IL, USA, Cy3-conjugated donkey anti-goat IgG 1:300; Jackson Immunoresearch Laboratories and Cy5-conjugated donkey anti-mouse IgG 1:300; Jackson Immunoresearch Laboratories) in 3% normal donkey serum with 0.3% Triton X-100 in 0.01 M PBS was applied to the sections, which were then incubated overnight at 4 °C. The sections were then washed, mounted on SuperFrost Plus slides (Menzel-Gläser), and coverslipped with medium containing 0.01 M PBS-buffered glycerol.

#### Quantitative evaluation of staining

For immunoenzymatic staining, the number of immunoreactive (BrdU^+^, *K*_i_-67^+^, DCX^+^ or NeuN^+^) cells in the DG of the hippocampus was estimated using unbiased stereological methods (West et al. 1991) following the procedure described by Maćkowiak et al. (Maćkowiak et al. 2007). Every ninth section along the rostrocaudal axis of the hippocampal formation was analysed using optical fractionator sampling, which was performed using a Leica DM 6000B light microscope equipped with a motorized stage (Ludl Electronic Products, Hawthorne, NY, USA) and digital camera (MBF C×9000, Williston, VT, USA). The dorsal DG was outlined under low magnification (2.5×) using Stereo Investigator software *v.* 8.0 (MBF Bioscience, Williston, VT, USA) according to a stereotaxic atlas of the rat brain (Paxinos and Franklin 2001). Sampling was performed bilaterally under high magnification (63×, oil-immersion objective) using counting frames with areas of 1600 µm^2^ (the analysis of BrdU^+^, *K*_i_-67^+^ or DCX^+^ cells) or 3600 (the analysis of NeuN^+^ cells) µm^2^ and heights of 15 μm. Cells appearing in the upper focal plane were omitted to prevent counting the tops of the cells (− 5 μm of the topmost surface of the section). The mean numerical density of immunoreactive cells was calculated for each animal from the sum of the counts made within the optical dissectors. The final results are presented as the density of immunoreactive cells in the DG of the hippocampus, calculated as the total number of cells estimated by optical fractionator divided by the estimated DG volume in mm^3^.

For immunofluorescent triple labelling, the colocalization of BrdU, DCX, and/or NeuN immunoreactivity in the sections was visualized using a confocal microscope (Leica TCS SP8, WLL) with excitation wavelengths of 495 nm (Alexa 488), 550 nm (Cy3), and 650 nm (Cy5). The sections were scanned using a 63× objective (HC PL APO CS2 63×/1.40 OIL) along the *Z*-axis (*Z*-step size: 1 μm, scan speed: 400 Hz, frame size: 512 × 512, line average: 3). *Z*-plane stacks of images were collected at every location within the hippocampal DG in which BrdU^+^ cells were visible. Images were further examined using Leica Application Suite X software (LAS X; Leica Microsystems, Switzerland) to observe the phenotype of the surviving cells. For each rat, the number of single-labelled cells (BrdU^+^ cells labelled for neither DCX nor NeuN), double-labelled cells (BrdU^+^ cells labelled for either DCX or NeuN; BrdU^+^/DCX^+^ or BrdU^+^/NeuN^+^, respectively) and triple-labelled cells (BrdU^+^ cells expressing both DCX and NeuN; BrdU^+^/DCX^+^/NeuN^+^) were estimated. The results are presented as the percentages of BrdU^+^ cells labelled for the respective marker(s) for each cell type in the given region.

#### Preparation of photomicrographs for data presentation

To present examples of cells that exhibited immunoreactivity for the neurogenesis markers used in the analysis, immunoenzymatically stained sections were imaged using a Leica MICA WideFocal Live Cell system (Leica Microsystems), while immunofluorescence staining was displayed in representative confocal microscopy images of *Z*-plane stacks from a control animal. Final photomicrographs were compiled with ImageJ *v.* 1.52q (NIH, Bethesda, MA, USA) and CorelDRAW Graphics Suite 2023 (Corel Corporation, Ottawa, Canada) software.

### Effects of long-term exercise during nicotine withdrawal on the behaviour of rats during nicotine deprivation

Separate groups of rats (*n* = 12) were generated through a self-administration procedure. After achieving stable responses on the active lever, the animals were subjected to an abstinence phase. Twenty-four hours after the last self-administration session, the rats were transferred to cages equipped with either running wheels (NIC– WR; *n* = 6) or locked wheels (NIC– LW; *n* = 6) until Day 14 of nicotine cessation.

#### The FST

On Day 13 of nicotine abstinence, the rats were individually placed in a nontransparent cylindrical tank (50-cm high, 23 cm in diameter) filled with water (30-cm deep, 25 ± 1 °C), and they remained there for 15 min (the pretest) (Zaniewska et al. 2021). The rats were then removed, dried, and returned to the appropriate home cages. On Day 14, immediately after the locomotor activity test, the rats underwent the FST for 5 min (the test). The following behavioural parameters were measured by two experimenters: immobility time, swimming and climbing. All the test sessions were recorded by a video camera to allow for repeated measurements. After the FST, all animals were returned to standard home cages without wheels.

#### Locomotor activity

On Day 14, after the final 24-h exposure to the running wheels (the 13th session), all animals were placed in standard home cages. Their locomotor activity was then assessed without prior habituation to the test environment (spontaneous locomotor activity) (Zaniewska et al. 2021). Activity was recorded in Opto-Varimex cages (Columbus Instruments, USA). Interruptions of the photobeams resulted in the measurement of horizontal locomotor activity, which was defined as the distance travelled (expressed in cm). Locomotor activity was recorded during 5- or 30-min trials. Subsequently, the animals were transferred to their standard home cages.

#### Nicotine-seeking behaviour

On Day 15, 24 h after the last running wheel exposure, the rats were administered nicotine (0.4 mg/kg, sc, unconditioned stimulus) and immediately introduced to the experimental cages to induce a drug-seeking response. During a 2-h session, pressing the active lever on an FR5 schedule resulted in the intravenous delivery of saline without conditioned cues (light + tone). Similar to the maintenance phase, a 20-s time-out period followed each infusion. The number of lever presses corresponds to presses made under the FR schedules, during infusions, and time-out periods. The strength of nicotine-seeking behaviour, as indicated by the number of active lever presses, was compared between animals that had access to unlocked running wheels and those with access to locked wheels.

### Statistical analyses

The data are expressed as means (± SEM). The sample size calculation was based on our preliminary behavioural (self-administration, FST) and neurobiochemical (hippocampal neurogenesis) data showing that to obtain statistically significant differences, a minimum of 6 (behavioural analyses) or 4–5 (neurogenesis analyses) rats per group is necessary. The normality of the data distribution was tested by the Shapiro‒Wilk normality test. After examining all the assumptions (i.e., normal distribution, equality of variance, existence of outliers), appropriate statistical tests were applied. Comparisons between means representing changes from the control values were made using Student’s *t* test for independent samples (cumulative nicotine intake/FST/locomotor activity). For non-normally distributed data, the Mann‒Whitney U test was used. Two-way analysis of variance (ANOVA) for repeated measures with interaction was used to analyse the lever presses during the maintenance phase across sessions 2 to 21 (factors: lever, treatment (nicotine), session) and body weight gain during nicotine abstinence (neurogenesis experiment). One-way ANOVA for repeated measures with interaction was applied to analyse the running distance (neurogenesis experiment) as well as lever presses during the maintenance phase (factors: lever, session) and body weight gain (factors: environment (locked wheels/running wheels), abstinence day) (behavioural experiment). Repeated measures ANOVA with interaction was used to assess nicotine infusions across sessions 2 to 21 (behavioural and neurogenesis experiments) and running distance (behavioural experiment). The animals’ body weight on the last self-administration day (factors: treatment (nicotine), environment), the immunostaining data (factors: treatment (nicotine deprivation), environment), and the number of lever presses taken during the induction of drug-seeking behaviour (factors: lever, environment) was analysed using two-way ANOVA with interaction. One-way ANOVA was used to analyse the cumulative nicotine intake in the neurogenesis experiment. ANOVA was followed by a post hoc Tukey test. All comparisons were made with an experiment type I error rate (α) set at *p* < 0.05. Statistics were calculated using GraphPad Prism v.9.3.0 software (GraphPad Software, La Jolla, CA, USA) or Statistica v. 13.3 (TIBCO Statistica).

## Results

### Rats acquired nicotine self-administration during 21 sessions

Rats that self-administered nicotine showed significant changes in the number of lever presses across sessions 2 to 21 (treatment × lever presses × session interaction (F(19,1368) = 9.28, *p* < 0.001, η^2^_p_ = 0.11), treatment (F(1,72) = 83.04, *p* < 0.001, η^2^_p_ = 0.54), lever (F(1,72) = 37.70, *p* < 0.001, η^2^_p_ = 0.34), and session (F(19,1368) = 14.85, *p* < 0.001, η^2^_p_ = 0.17)). Post hoc Tukey analysis revealed that the number of active lever presses in nicotine self-administering rats was significantly greater than inactive lever presses during sessions 9–10 (*p* < 0.05) and 11–21 (*p* < 0.05), and significantly higher than active lever presses in control rats during sessions 8 (*p* < 0.05) and 9–21 (*p* < 0.001) (Fig. 1b). The number of active lever presses during sessions 9–10 (*p* < 0.05) and 11–21 (*p* < 0.001) was also higher than active lever presses during session 2 (Fig. 1b).

The number of nicotine infusions varied across sessions (F(19,361) = 1.99, *p* < 0.01, η^2^_p_ = 0.095), but post hoc Tukey analysis revealed no significant differences in the number of infusions in the last sessions (17–21; *p* > 0.05; Fig. 1c). The total nicotine intake did not vary among the three assigned groups (exposed to running wheels, locked wheels or housed in home cages during nicotine cessation) (F(2,17) = 0.0072, *p* = 0.99, η^2^_p_ = 0.00085; Fig. 1d).

### Nicotine deprivation increased running distance

Nicotine deprivation did not affect running distance by abstinence days (treatment × abstinence day interaction (F(12,144) = 1.36, *p* = 0.19, η^2^_p_ = 0.10)), but there were significant effects of treatment (F(1,12) = 5.22, *p* = 0.041, η^2^_p_ = 0.30) and abstinence days (F(12,144) = 6.22, *p* < 0.001, η^2^_p_ = 0.34). Post hoc analysis revealed that the running distance on abstinence Day 14 was significantly greater than on Day 2 (*p* = 0.0029; Fig. 1e). Over 13 days, nicotine-deprived rats ran significantly more than saline-treated rats (YSAL– WR: 10.65 ± 1.41 km; NIC– WR: 18.95 ± 3.35 km; *t* = 2.29, df = 12, *p* = 0.041, *d* = 1.22).

### Voluntary wheel running reduced body weight gain in rats compared to those in home cages

On the last day of self-administration, there were no differences in body weight among the treatment conditions (treatment × environment interaction (F(2,32) = 0.21, *p* = 0.81, η^2^_p_ = 0.013)), nor was there an effect of treatment (F(1,32) = 0, *p* = 0.99, η^2^_p_ = 0.000009) or environment (F(2,32) = 0.78, *p* = 0.47, η^2^_p_ = 0.047) (Fig. 1f). Nicotine deprivation did not significantly change the body weight according to the environmental conditions or duration of abstinence (treatment × environment × abstinence day interaction (F(6,96) = 1.06, *p* = 0.39, η^2^_p_ = 0.062), treatment (F(1,32) = 0.011, *p* = 0.92, η^2^_p_ = 0.00035); Fig. 1f). However, significant effects of environment (F(2,32) = 5.56, *p* = 0.0085, η^2^_p_ = 0.26) and abstinence day (F(3,96) = 322.46, *p* < 0.001, η^2^_p_ = 0.91) on body weight were observed. Post hoc Tukey tests showed that rats with running wheels had significantly lower body weights than home-caged rats (*p* = 0.0099; Fig. 1f), with a nonsignificant trend for reduced weight compared to locked-wheel rats (*p* = 0.066; Fig. 1f). Over the course of abstinence, the animals gained weight, and the body weight on the final day was significantly greater than on the previous days (*p* = 0.00014; Fig. 1f).

### Voluntary wheel running increases cell proliferation in the rat DG

**Fig. 2:**
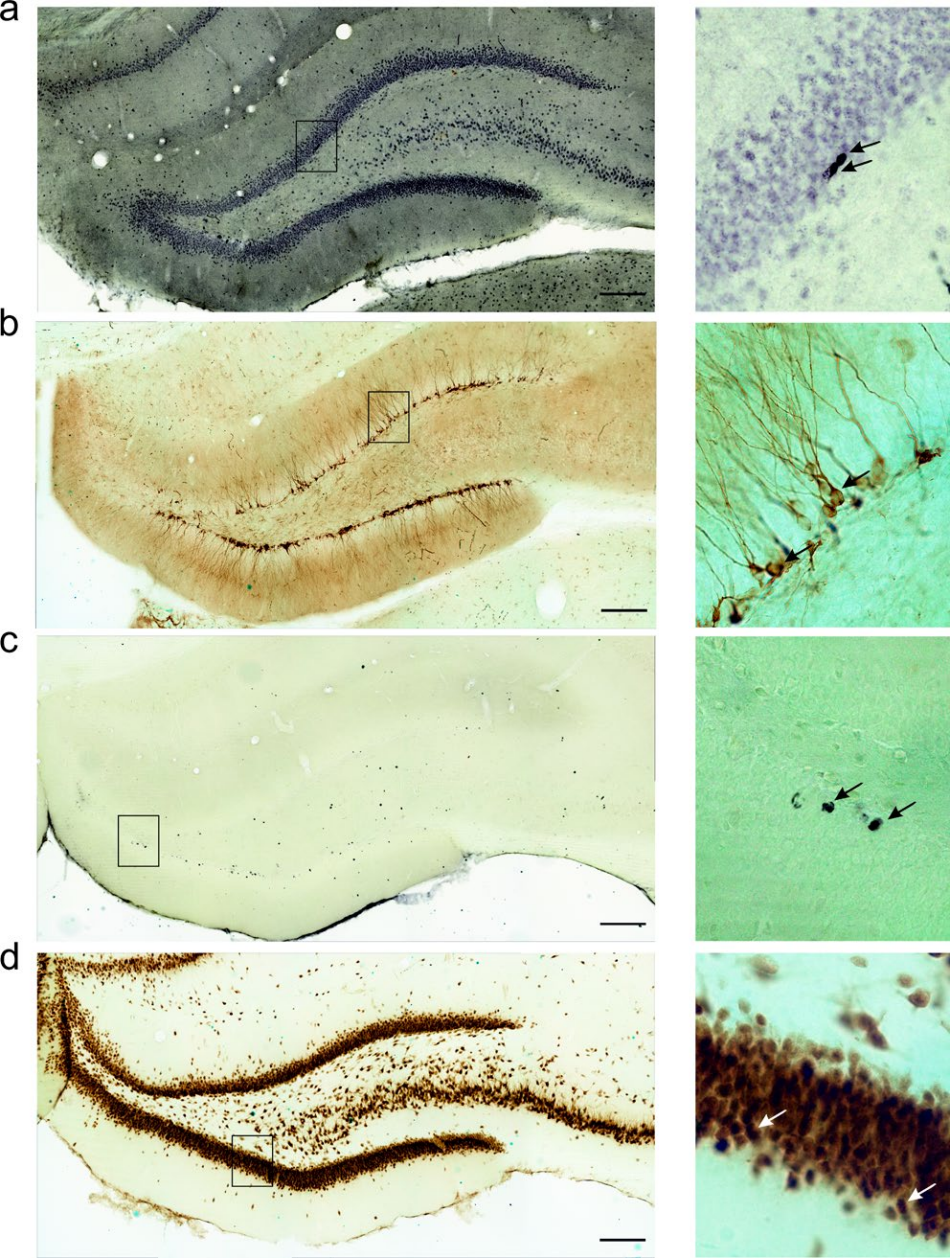
Immunoenzymatic staining of newborn cells in the hippocampal dentate gyrus (DG) during nicotine deprivation. Representative photomicrographs of cells labelled with *K*_i_-67 (a proliferation marker; (**a**)), doublecortin (DCX, a marker of immature neurons; (**b**)), BrdU (a marker of survival of proliferating cells; (**c**)), and NeuN (a marker of adult neurons; (**d**)) (indicated by the black or white arrows) in control animals that received saline infusions (YSAL) and were housed in home cages during the deprivation period (YSAL–home). The images in the left panels were captured under a 20× magnification objective lens, while examples of individual cells (using a 63× objective) are depicted in the right panels. Scale bars: 200 μm

**Fig. 3:**
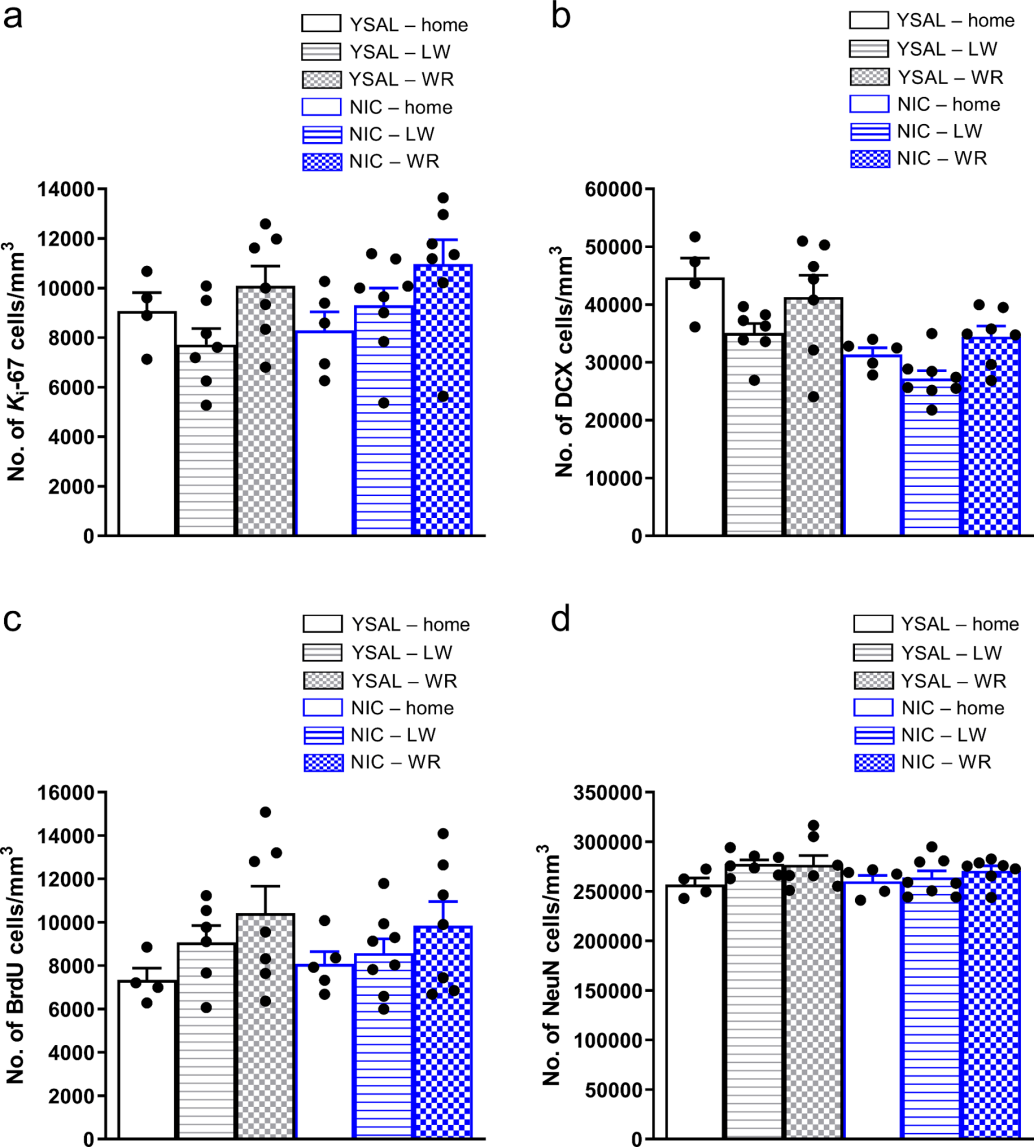
Effects of wheel-running exercise on the number of newborn cells in the hippocampal dentate gyrus (DG) during nicotine (NIC) deprivation (Day 14). Effects of wheel running on the total number of (**a**) proliferating (*K*_i_-67^+^) cells/mm^3^, (**b**) immature (DCX^+^) cells/mm^3^, (**c**) surviving (BrdU^+^) cells/mm^3^, and (**d**) neuronal (NeuN^+^) cells/mm^3^ in the DG during NIC cessation. The data are expressed as the means (± SEM). YSAL–home: *n* = 4; YSAL–LW: *n* = 7; YSAL–WR: *n* = 7; NIC–home: *n* = 5; NIC–LW: *n* = 8; NIC–WR: *n* = 7. (**a**) *p* < 0.05: the effect of environment: post hoc Tukey: *p* < 0.05: WR vs. LW. (**b**) *p* < 0.001: the effect of NIC deprivation; *p* < 0.01: the effect of environment: post hoc Tukey: *p* < 0.05: LW vs. home, *p* < 0.01: WR vs. LW. For further details, please see the caption of Fig. 1

Nicotine deprivation did not change the number of *K*_i_-67^+^ cells (Figs. 2a and 3a) based on the environmental conditions in which the animals were kept during drug deprivation (treatment × environment interaction (F(2,32) = 0.98, *p* = 0.39, η^2^_p_ = 0.057), treatment (F(1,32) = 0.69, *p* = 0.41, η^2^_p_ = 0.021)). However, the environment significantly affected cell proliferation (F(2,32) = 4.17, *p* = 0.025, η^2^_p_ = 0.21, Fig. 3a). Post hoc analysis showed that rats exposed to running wheels exhibited significantly greater proliferation than did those with locked wheels (*p* = 0.035); running induced ca. a 31% increase in cell proliferation in saline-treated animals and an 18% increase in nicotine-deprived rats (Fig. 3a). Overall, these findings indicated that wheel running has a positive impact on cell proliferation in the DG of rats.

### Wheel running increases the number of immature neurons in the rat DG

Wheel running did not affect the number of DCX^+^ cells (Figs. 2b and 3b) in nicotine-deprived rats (treatment × environment interaction (F(2,32) = 0.91, *p* = 0.41, η^2^_p_ = 0.054)), but nicotine deprivation (F(1,32) = 22.82, *p* = 0.000038, η^2^_p_ = 0.42) and environment (F(2,32) = 6.08, *p* = 0.0058, η^2^_p_ = 0.28) significantly influenced the number of DCX^+^ cells. Post hoc Tukey test for the effect of the environmental factor showed that rats exposed to locked wheels had fewer DCX^+^ neurons than those kept in home cages (*p* = 0.037; Fig. 3b). Rats exposed to running wheels exhibited an increased number of immature neurons compared to animals with locked wheels (*p* = 0.0083); running induced ca. an 18% increase in the number of DCX^+^ neurons in saline-treated rats and a 33% increase in nicotine-deprived rats (Fig. 3b). Thus, wheel running enhances maturation of new neurons, while the exposure to cages with locked running wheels and nicotine cessation have negative effects on the maturation of new neurons.

### Voluntary wheel running does not alter the survival of newborn cells during nicotine cessation

Chronic wheel running did not affect the number of BrdU^+^ cells in the DG two weeks post-BrdU labelling (Figs. 2c and 3c) (treatment × environment interaction (F(2,31) = 0.25, *p* = 0.78, η^2^_p_ = 0.016)). There was no effect of treatment (F(1,31) = 0.02, *p* = 0.89, η^2^_p_ = 0.00064) or environment (F(2,31) = 3.02, *p* = 0.063, η^2^_p_ = 0.16) on the number of BrdU^+^ cells (Fig. 3c), indicating that the survival of newly generated cells in nicotine-deprived rats is not altered by chronic wheel running.

### Voluntary wheel running does not change the number of adult-born neurons during nicotine cessation

Wheel running did not affect the number of adult-born neurons (NeuN^+^; Figs. 2d and 3d) (treatment × environment interaction (F(2,32) = 0.67, *p* = 0.52, η^2^_p_ = 0.040). No significant effects were observed for treatment (F(1,32) = 0.92, *p* = 0.34, η^2^_p_ = 0.028) or environment (F(2,32) = 2.32, *p* = 0.11, η^2^_p_ = 0.13) on the number of NeuN^+^ cells, suggesting that chronic wheel running does not alter the number of adult-born neurons in nicotine-deprived rats.

### Voluntary wheel running does not affect the phenotype of surviving cells during nicotine cessation

**Fig. 4:**
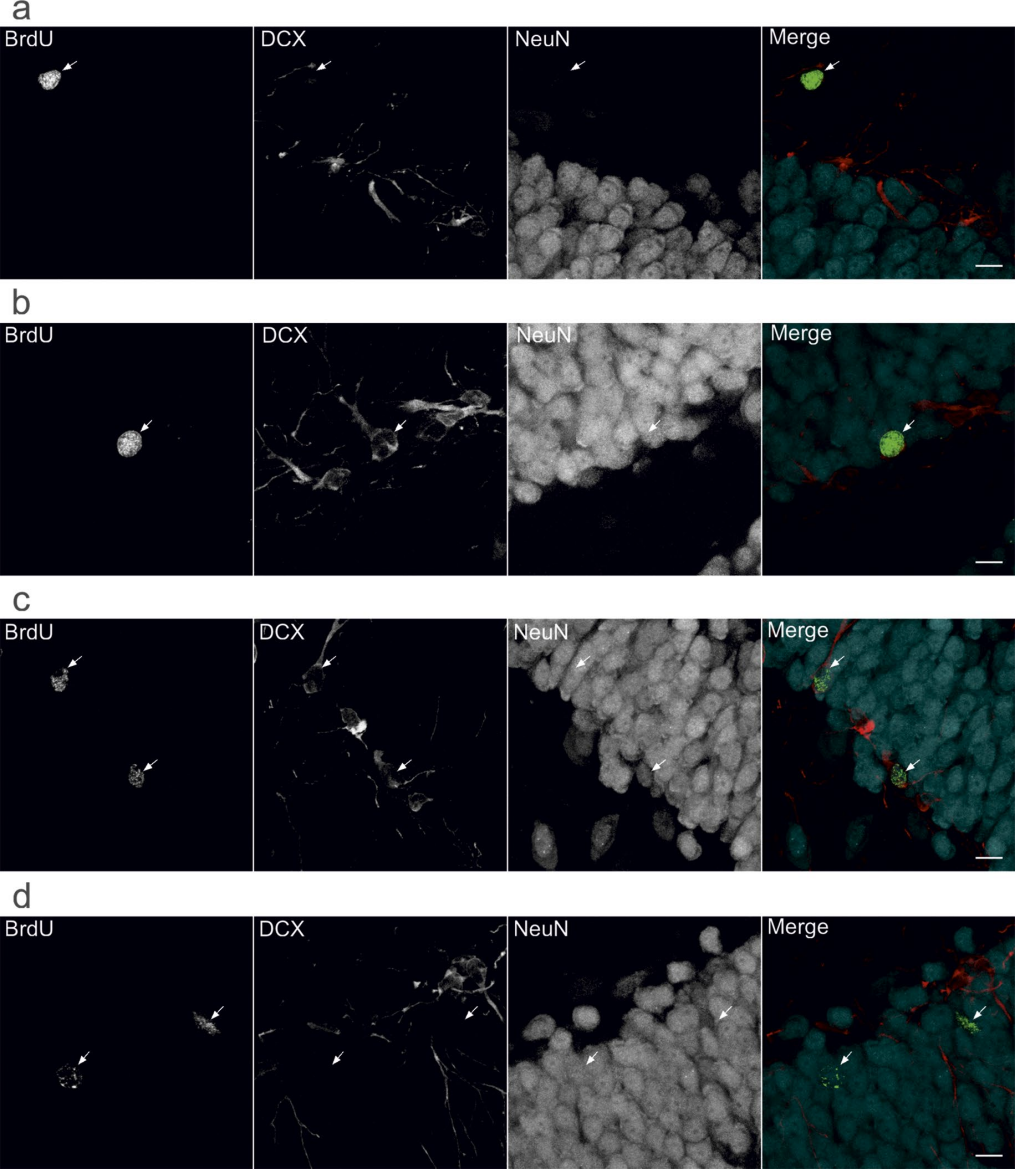
Colocalization studies of surviving cells in the hippocampal dentate gyrus (DG) during nicotine deprivation. Immunofluorescent triple labelling was used to determine changes in the phenotype of the surviving cells. The brain sections containing the proximal and distal parts of the hippocampus were costained with BrdU (a marker of survival of proliferating cells; green label), DCX (a marker of immature neurons; red label), and NeuN (a neuronal marker; cyan label). (**a**–**d**) Representative confocal microscopy images of *Z*-plane stacks showing single-labelled (BrdU^+^; **a**), immature neuronal (double-labelled (BrdU^+^/DCX^+^; **b**) or triple-labelled (BrdU^+^/DCX^+^/NeuN^+^; **c**)), and adult neuronal (double-labelled (BrdU^+^/NeuN^+^; **d**)) cells (white arrows) in control animal that received saline infusions (YSAL) and was housed in its home cage during the deprivation period (YSAL–home). Scale bars: 10 μm

**Fig. 5:**
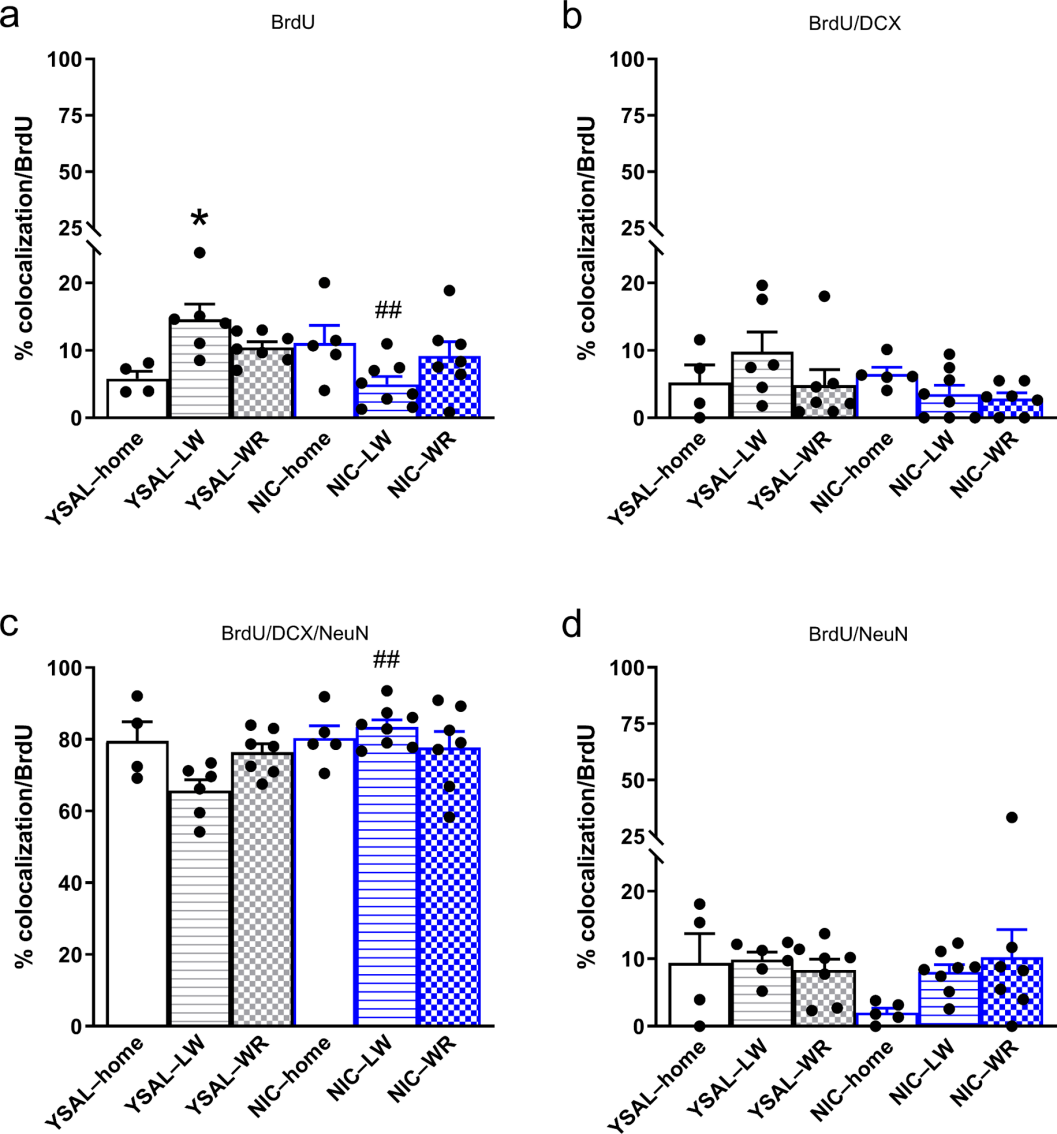
Effects of wheel-running exercise on the differentiation of surviving cells in the hippocampal dentate gyrus (DG) during nicotine (NIC) deprivation (Day 14). Immunofluorescent triple labelling was used to determine changes in the phenotype of the surviving cells. The brain sections containing the proximal and distal parts of the hippocampus were costained with BrdU (a marker of survival of proliferating cells), DCX (a marker of immature neurons), and NeuN (a neuronal marker). (**a**–**d**) The effects of wheel running exercise on different cell types: single-labelled (BrdU^+^; **a**), immature neuronal (double-labelled (BrdU^+^/DCX^+^; **b**) or triple-labelled (BrdU^+^/DCX^+^/NeuN^+^; **c**)) and adult neuronal (double-labelled (BrdU^+^/NeuN^+^; **d**)) cells during NIC cessation. Cell types were assessed by calculating the percentage of BrdU^+^ cells labelled with the respective marker(s) for each cell population. The results are expressed as means (± SEM). YSAL–home: *n* = 4; YSAL–LW: *n* = 7; YSAL–WR: *n* = 7; NIC–home: *n* = 5; NIC–LW: *n* = 8; NIC–WR: *n* = 7. (**a**) Post hoc Tukey: ^*^p < 0.05 vs. YSAL–home, ^##^*p* < 0.01 vs. YSAL–LW. (**c**) Post hoc Tukey: ^##^*p* < 0.01 vs. YSAL–LW. For further details, please see the caption of Fig. 1

The analysis of the phenotype of surviving cells (Figs. 4 and 5) revealed that the percentage of single-labelled (BrdU^+^) cells (Fig. 4a) was significantly affected by environmental factors (treatment × environment interaction (F(2,31) = 8.39, *p* = 0.0012, η^2^_p_ = 0.35). There was no effect of treatment (F(1,31) = 1.61, *p* = 0.21, η^2^_p_ = 0.049) or environment (F(2,31) = 0.32, *p* = 0.73, η^2^_p_ = 0.020) on the percentage of BrdU^+^ cells (Fig. 5a). Post hoc Tukey tests showed that the percentage of BrdU^+^ cells was greater in rats that received saline infusions and were exposed to locked wheels compared to control animals (*p* = 0.039; Fig. 5a). In nicotine-deprived rats, the percentage of BrdU^+^ cells was lower in those with locked wheels than in rats that received saline infusions and were housed in similar conditions (*p* = 0.0034; Fig. 5a). The BrdU^+^ cell populations in nicotine-deprived and saline-treated animals housed in standard cages did not differ (*p* = 0.46; Fig. 5a).

Environmental factors significantly affected the percentage of surviving cells labelled for both DCX and NeuN (BrdU^+^/DCX^+^/NeuN^+^; Figs. 4c and 5c) (treatment × environment interaction (F(2,31) = 4.30, *p* = 0.022; η^2^_p_ = 0.22)). There was also a significant effect of treatment (F(1,31) = 5.56, *p* = 0.025, η^2^_p_ = 0.15), but no effect of environment was reported (F(2,31) = 1.14, *p* = 0.33, η^2^_p_ = 0.068). Post hoc analysis showed that housing nicotine-deprived animals in cages with locked wheels enhanced the percentage of immature neurons (BrdU^+^/DCX^+^/NeuN^+^) (by 27%) compared to saline controls housed in similar conditions (*p* = 0.0051; Fig. 5c). There was no distinction in the BrdU^+^/DCX^+^/NeuN^+^ population in nicotine-deprived animals versus saline animals housed under standard conditions (*p* > 0.05; Fig. 5c).

Environmental conditions had no effect on the percentage of BrdU^+^ cells expressing DCX (BrdU^+^/DCX^+^; Figs. 4b and 5b) (treatment × environment interaction (F(2,31) = 1.76, *p* = 0.19, η^2^_p_ = 0.10), treatment (F(1,31) = 2.10, *p* = 0.16, η^2^_p_ = 0.064), environment (F(2,31) = 1.28, *p* = 0.29, η^2^_p_ = 0.076)) or NeuN (BrdU^+^/NeuN^+^; Figs. 4d and 5d) (treatment × environment interaction (F(2,31) = 1.55, *p* = 0.23; η^2^_p_ = 0.091), treatment (F(1,31) = 1.36, *p* = 0.25, η^2^_p_ = 0.042), environment (F(2,31) = 1.06, *p* = 0.36, η^2^_p_ = 0.064)) during nicotine deprivation.

### Nicotine self-administration in rats selected for behavioural analyses

**Fig. 6:**
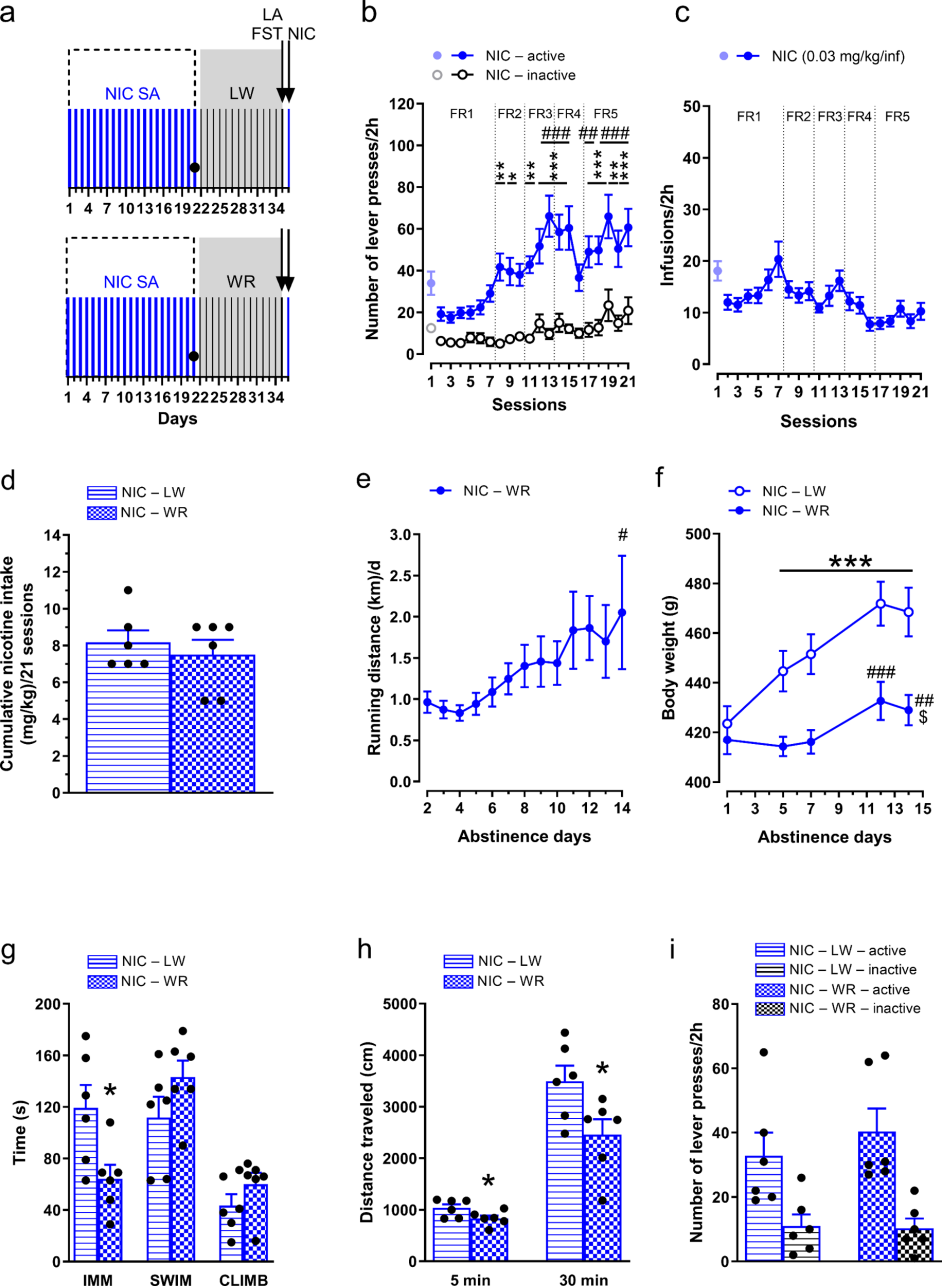
Effects of wheel-running exercise on behavioural symptoms of nicotine deprivation. Rats were allowed to self-administer nicotine (0.03 mg/kg/inf, NIC SA) in 2-h sessions. After 21 sessions, the rats entered the deprivation phase for 14 days. On abstinence Day 1, the animals were transferred to cages equipped with running wheels (WR) or locked wheels (LW) for the next 13 abstinence days. The following behavioural responses were measured during NIC deprivation: depression-like behaviour in the forced swim test (FST; abstinence Day 14), locomotor responses (LA; abstinence Day 14), and nicotine-seeking behaviour induced by NIC priming (0.4 mg/kg, sc; abstinence Day 15). (**a**) The experimental schedule for NIC SA and WR exposure during NIC cessation, followed by behavioural analyses. (**b**) The number of lever presses (made under the fixed ratio (FR) schedule, during infusions, and the time-out period) in rats (*n* = 12) self-administering NIC during an increasing schedule of reinforcement (FR (1–5)). The lever presses in session 1 were shown; however, they were not included in the analysis due to the effect of previous water training on lever pressing on this day. (**c**) NIC infusions in rats (*n* = 12) throughout 21 self-administration sessions. The infusions in session 1 were not included in the analysis due to the effect of previous water training on infusions on this day. (**d**) Cumulative NIC intake (mg/kg) during 21 sessions in the two assigned groups (exposed to WR or LW during NIC cessation; *n* = 6 rats/group). (**e**) Daily running distance ((km)/d) in the NIC-deprived group that had access to WR (*n* = 6). (**f**) Effects of WR on the body weight (**g**) of rats deprived of NIC (*n* = 6 rats/group). (**g**) Effects of WR on the behaviour of rats (*n* = 6 rats/group) in the FST during the long-term NIC deprivation period. The following parameters were measured: immobility (IMM) time (s), swimming (SWIM) time, and climbing (CLIMB) time. (**h**) Effects of WR on LA during drug cessation (*n* = 6 rats/group). (**i**) Effects of WR on lever presses (made under the FR schedule, during infusions, and the time-out period) induced by NIC priming during abstinence (*n* = 6 rats/group). The data are expressed as the means (± SEM). (**b**) Post hoc Tukey test: ^*^p < 0.05, ^**^p < 0.01, ^***^p < 0.001 vs. inactive lever presses; ^##^*p* < 0.01, ^###^*p* < 0.001 vs. active lever presses on session 2. (**e**) Post hoc Tukey: ^#^*p* < 0.05 vs. abstinence Day 2. (**f**) Post hoc Tukey test: ^***^p < 0.001 vs. abstinence Day 1 (NIC–LW); ^##^*p* < 0.01, ^###^*p* < 0.001 vs. abstinence Day 1 (NIC–WR); ^$^*p* < 0.05 vs. NIC–LW. (**g**) *t*-test: ^*^p < 0.05 vs. NIC–LW. (**h**) *t*-test: ^*^p < 0.05 vs. NIC–LW. (**i**) *p* < 0.001: the effect of lever: active vs. inactive lever presses

Nicotine intake in the behavioural experiment (Fig. 6 a) was comparable to that reported in the analysis of hippocampal neurogenesis. Nicotine altered lever pressing across sessions (lever presses × session interaction (F(19,418) = 4.22, *p* < 0.001, η^2^_p_ = 0.16), lever presses (F(1,22) = 47.53, *p* < 0.001, η^2^_p_ = 0.68), session (F(19,418) = 10.66, *p* < 0.001, η^2^_p_ = 0.33)). Post hoc Tukey analysis indicated more active than inactive lever presses in rats self-administering nicotine during sessions 8, 11, and 20 (*p* < 0.01); session 9 (*p* < 0.05); and sessions 12–15, 17–19, and 21 (*p* < 0.001) (Fig. 6b). The number of active lever presses during sessions 12–15, 18–21 (*p* < 0.001) and 17 (*p* < 0.01) was significantly greater than during session 2 (Fig. 6b).

The number of nicotine infusions varied across sessions 2 to 21 (F(19,209) = 4.36, *p* < 0.001, η^2^_p_ = 0.28; Fig. 6c). However, post hoc Tukey analysis showed that no significant differences were found between infusions during the last sessions (17–21) (*p* > 0.05; Fig. 6c). Total nicotine intake was similar between the two assigned (exposed to running wheels or locked wheels) groups (U = 17, *p* > 0.05; Fig. 6d).

### Exposure to running wheels increases the running distance in nicotine-deprived rats

The running distance of nicotine-deprived animals changed significantly over 13 days of nicotine abstinence (Days 2–14) (F(12,60) = 3.72, *p* = 0.00033, η^2^_p_ = 0.43; Fig. 6e). Post hoc Tukey analysis showed that the distance on abstinence Day 14 was significantly greater than that on Day 2 (*p* = 0.032; Fig. 6e).

### Voluntary wheel running during nicotine deprivation reduces body weight gain

Voluntary wheel running during drug cessation significantly changed the body weight gain (environment × abstinence day interaction (F(4,40) = 22.74, *p* < 0.001, η^2^_p_ = 0.69), environment (F(1,10) = 9.31, *p* = 0.012, η^2^_p_ = 0.48), abstinence day (F(4,40) = 86.44, *p* < 0.001, η^2^_p_ = 0.90); Fig. 6f)). Post hoc Tukey tests showed that rats self-administering nicotine and exposed to locked wheels during drug cessation had greater body weight on abstinence Days 5–14 compared to Day 1 (*p* < 0.001; Fig. 6f). In the running group, the body weight on abstinence Days 12 and 14 was significantly greater than on Day 1 (*p* < 0.001 and *p* < 0.01, respectively; Fig. 6f). However, on the last day of abstinence, the body weight of running rats was lower (*p* = 0.049) than that of locked-wheel rats (Fig. 6f), suggesting that wheel runners exhibited less weight gain than rats with locked wheels.

### Wheel running reduces immobility time in the forced swim test during nicotine cessation

In the FST, wheel running significantly affected immobility time (t = 2.63, df = 10, *p* = 0.025, *d* = 1.52) but had no effect on swimming (t = 1.52, df = 10, *p* = 0.16, *d* = 0.88) or climbing (U = 10, *p* = 0.22) during long-term nicotine deprivation (Day 14; Fig. 6g).

### Voluntary wheel running reduces locomotor activity during nicotine cessation

On abstinence Day 14, rats exposed to the running wheels showed significantly reduced spontaneous motor activity during the 5-min (t = 2.23, df = 10, *p* = 0.0497, *d* = 1.29; Fig. 6h) and 30-min (t = 2.42, df = 10, *p* = 0.036, *d* = 1.40; Fig. 6h) observation periods.

### Voluntary wheel running does not alter drug-seeking behaviour during nicotine abstinence

On abstinence Day 15, there were no significant differences in the number of active lever presses (made under the FR schedule, during infusions, and during the time-out period) between rats with locked and unlocked running wheels during nicotine cessation (lever press × environment interaction (F(1,20) = 0.53, *p* = 0.47, η^2^_p_ = 0.026), environment (F(1,20) = 0.37, *p* = 0.55, η^2^_p_ = 0.018)). A significant main lever effect was observed (F(1,20) = 21.36, *p* = 0.00017, η^2^_p_ = 0.52), indicating more active than inactive lever presses in both treatment groups (*p* < 0.001; Fig. 6i). As outlined in the Supplementary file (Suppl. Figure 1), no differences were observed in the number of lever presses between the groups when analysed separately for each phase of the drug-seeking session.

## Discussion

This study demonstrated that voluntary exercise (wheel running) had beneficial effects on the initial stages of hippocampal neurogenesis (proliferation and maturation of developing neurons) in both nicotine-deprived and healthy rats. Compared with saline, long-term exercise increased the running distance of nicotine-deprived rats. Additionally, wheel running exerted an antidepressant effect on rats subjected to nicotine deprivation without influencing nicotine-seeking behaviour.

The present investigation confirmed a pattern of nicotine self-administration similar to our previous findings (Zaniewska et al. 2021), showing an increase in the number of active lever presses in rats self-administering nicotine under the increasing reinforcement schedule, indicating continued motivation for nicotine in animals. In our study, the rats self-administered ca. 0.38 mg/kg/day nicotine, which is consistent with earlier studies on limited-access nicotine (Irvine et al. 2001; Cohen et al. 2015; Zaniewska et al. 2021).

### Effects of voluntary exercise on physical health parameters in rats

In this research, we observed amplified running distances in both control and nicotine-deprived animals at the end of drug deprivation compared to the initial day, with control rats running less (0.82 km/day) than reported in prior studies using the same rat strain (2.8 km/day) (Kodali et al. 2016). Preexposure procedures, including surgery or saline infusions, may have influenced the animals’ motor activity. Nicotine-deprived animals ran significantly more (19 km over 13 days) than controls (11 km), suggesting an enhanced need for physical activity during nicotine cessation, a finding consistent with data on tobacco smokers abstaining from smoking (Ussher et al. 2003; Harrison et al. 2019). In contrast, such an increase in activity was not found in adolescent rats during a shorter 10-day period of nicotine deprivation with limited (2-h) daily exercise (Sanchez et al. 2013), suggesting the importance of exercise duration for running activity. It remains to be determined whether nicotine withdrawal or other factors, such as deprivation of operant behaviour(Fouyssac et al. 2022) were responsible for the increased running in nicotine-deprived animals.

During a 2-week voluntary exercise period, both nicotine-deprived and saline-treated animals exhibited less body weight gain than did those in locked-wheel or standard cages, indicating the role of wheel-running exercise in weight management. This finding aligns with a clinical study showing less weight gain in vigorously exercising abstinent smokers (Marcus et al. 1999), although another study did not confirm such an effect (Harrison et al. 2019). The body weight reduction in our Sprague‒Dawley rats may have been due to exercise-induced transient hypophagia and increased liquid intake, as observed in previous research (Droste et al. 2007).

### Effects of nicotine deprivation and voluntary exercise on hippocampal neurogenesis

Consistent with prior findings (Zaniewska et al. 2021), this study revealed a decrease in the number of immature neurons (DCX^+^) in the hippocampal DG of nicotine-deprived rats without affecting cell proliferation (*K*_i_-67^+^). No significant alterations in cell differentiation or survival (BrdU^+^) were found. Although there was a 7% reduction in the number of newborn cells differentiating into adult neurons (BrdU^+^/NeuN^+^) and a 5% increase in the number of single-labelled (BrdU^+^) cells in nicotine-deprived animals, these fluctuations were not statistically significant. These results diverge from a previous report of reduced cell survival and differentiation upon nicotine cessation (Zaniewska et al. 2021), possibly due to different experimental setups, including stress from chronic drug injections in earlier study, which could impact neurogenesis. Research indicates that chronic stress can lead to a decrease in the survival and differentiation of newborn neurons in the rat DG (Lee et al. 2006; Veena et al. 2009; Dagytė et al. 2011; Alves et al. 2018), suggesting that additional stressors might influence the magnitude of neurogenesis alterations during nicotine cessation.

Although nicotine deprivation led to a decrease in the number of immature neurons, the number of adult-born neurons (NeuN^+^) in the hippocampal region remained unchanged. The two-week study period may not have been sufficient for capturing the full extent of changes in neurogenesis, as it typically takes ca. 4 weeks for new neurons to mature (Van Praag et al. 2002; Espósito et al. 2005). Future research extending to 4 weeks post-nicotine cessation is necessary to better understand the impact of reduced numbers of immature neurons on the overall population of neurons in the hippocampus.

Previous studies have shown that limited (1-h) nicotine access leading to daily intake between 0.18 and 0.32 mg/kg, decreased neurogenesis and expression of PSA-NCAM, and increased cell death in the DG (Abrous et al. 2002). Prolonged nicotine exposure (0.2–0.25 mg/kg/day) reduced the number of mature neurons (BrdU^+^/NeuN^+^) and the survival of new cells in the DG (Wei et al. 2012). Short-term deprivation of self-administered nicotine (ca. 0.3 mg/kg/day) did not affect neurogenesis markers (i.e., *K*_i_-67 or NeuroD, a marker of immature neurons) (Cohen et al. 2015). Conversely, rats with extended (21-h) access to nicotine (0.9 mg/kg daily) and periodic (3-day) deprivation displayed increased immature neuron counts (NeuroD^+^) in the SGZ of the DG and, after a short (75-h) period of withdrawal, enhanced cell proliferation (Cohen et al. 2015). These findings, along with our findings on late-stage nicotine withdrawal, demonstrate that nicotine consumption and abstinence duration induce complex effects on hippocampal neurogenesis, particularly affecting neuron maturation and migration. More research is needed to gain a comprehensive understanding of the functional relevance of these changes in neuroplasticity.

Our study showed that exercise increased cell proliferation and the number of immature neurons in the DG in both nicotine-deprived and control animals compared to those in animals with locked wheels. This increase in the early stages of hippocampal neurogenesis resulting from wheel running was not specific to nicotine deprivation but rather was a result of its beneficial effect on baseline neurogenesis. Importantly, in nicotine-deprived rats, the increase in immature neurons induced by running did not reach control levels, suggesting that exercise may be less effective under pathological conditions. This finding is consistent with findings from mice with complete 5-HT depletion in the brain, where impaired exercise-induced neurogenesis was demonstrated (Klempin et al. 2013). Two weeks of exposure to running wheels did not alter cell survival, differentiation, or the total number of mature neurons in the DG of drug-weaned rats at two weeks post-BrdU labelling. Notably, nicotine deprivation significantly altered the response of young neurons to large cages with locked-wheel access—a type of enriched environment—resulting in disrupted differentiation of surviving cells in the hippocampal DG compared to those in rats receiving saline infusions and housed under similar environmental conditions. These findings suggest that the loss of the beneficial effect of an enriched environment in nicotine-deprived rats possibly diminished the impact of wheel running (enriched environment + exercise) on deficits in hippocampal neurogenesis.

Research on C57BL/6 mice indicated that voluntary wheel running from 3 days to 2 weeks increased cell proliferation, and durations from 10 days to 8 weeks increased the number of immature neurons (DCX^+^) in the DG (Clark et al. 2010; Dostes et al. 2016; Fuss et al. 2010; Kronenberg et al. 2006; Nishijima et al. 2017; Ransome and Turnley 2008; Rich et al. 2017; Snyder et al. 2009; Van Praag et al. 1999). Running for more than 3 weeks increased newborn cell survival, with a greater percentage of neuronal BrdU^+^ cells (Brandt et al. 2003; Clark et al. 2008, 2010; Dostes et al. 2016; Mustroph et al. 2012; Snyder et al. 2009; Van Praag et al. 1999). Even 7 days of running effectively increased cell survival in C57BL/6 mice (Ransome and Turnley 2008), as assessed 4 weeks after BrdU labelling. Similar positive effects of running on hippocampal neurogenesis have been confirmed in other mouse strains (Clark et al. 2011; Merritt and Rhodes 2015) and rats, with varying periods of voluntary running boosting cell proliferation and increasing the number of DCX^+^ cells (Mukuda and Sugiyama 2007; Motta-Teixeira et al. 2016; Kodali et al. 2016). Running for one month or more in Sprague‒Dawley rats has been shown to increase new neuron survival and differentiation (Farmer et al. 2004; Kodali et al. 2016). Our findings are in line with these observations, indicating that running stimulates all stages of hippocampal neurogenesis. However, the lack of significant changes in cell survival and differentiation observed in our study may be due to the relatively short two-week interval studied here, which may have limited our ability to observe the full developmental progression of new neurons.

### Effects of voluntary exercise on nicotine cessation behaviours: recent studies and clinical perspectives

In the FST, nicotine-deprived rats with wheel access exhibited a shorter immobility time than those with locked wheels, suggesting an antidepressant effect of exercise. The wheel runners had lower spontaneous locomotor activity than did the sedentary animals, indicating that the reduction in immobility was specific and distinct from a general increase in arousal. Notably, our study did not include a non-nicotine-treated control group, limiting comparisons to drug-naive rats, i.e., evaluation of whether animals housed in locked-wheel cages developed depression-like behaviour. Nevertheless, our prior research indicated that nicotine-deprived rats housed in standard cages throughout the experiment exhibited depression-like behaviours compared to saline-treated controls (Zaniewska et al. 2021). In the current study, immobility times in nicotine-deprived rats with locked wheels were comparable to those observed in standard-housed rats, suggesting that the depression-like phenotype was also present in the locked-wheel group and was potentially mitigated by running in the wheel-access group.

Preclinical studies have shown that chronic wheel running has antidepressant effects on rodents, including Sprague‒Dawley rats (Chen et al. 2016) and Swiss mice (Cunha et al. 2013), but not on C57BL/6 mice (Morgan et al. 2018). It has been effective in animal models of depression, such as rats genetically prone to depression (Flinders sensitive line rats) and C57BL/6 mice under chronic stress (Bjørnebekk et al. 2005; Huang et al. 2017). Forced exercise on a treadmill during cessation from passive nicotine induced an antidepressant effect in the FST (Motaghinejad et al. 2016). In humans, running has improved depression symptoms (in an 8-week running program; (Doyne et al. 1987) and offered benefits comparable to those of antidepressants, along with additional physical health improvements (Verhoeven et al. 2023), suggesting its potential as an adjunctive intervention for depressive disorder (Kruisdijk et al. 2012). However, in smokers who quit tobacco, exercise does not improve depression symptoms (Patten et al. 2017; Harrison et al. 2019), although the specific benefits of running in this context have not been fully explored. Our findings, alongside these studies, underscore the potential of running as a therapeutic tool for depression, particularly for tobacco smokers with a history of depression.

Wheel-running exercise positively influenced mood but had no effect on nicotine-seeking behaviour in this study. Using an approach similar to that used in previous research, we induced drug-seeking behaviour after two weeks of nicotine abstinence by reintroducing nicotine and placing animals in an environment associated with earlier nicotine use (context + drug).

This study is the first to examine the effects of wheel running on nicotine seeking in adult rats. In adolescent male rats, limited (2-h) wheel running sessions decreased the acquisition of nicotine self-administration and the motivation to obtain nicotine (Sanchez et al. 2015). Restricted wheel running during a 10-day period of abstinence in male adolescents reduced nicotine seeking during extinction (context-induced drug seeking) but did not affect cue-induced reinstatement (Sanchez et al. 2013, 2014). In female adolescents, exercise decreased the extinction response and cue-primed reinstatement specifically during the oestrous phase (Lynch et al. 2019). Further research in adult rats is needed to determine whether the effects of running on nicotine-seeking behaviour are influenced by hormonal factors rather than age or exercise duration. Based on the findings of Fouyssac et al. (Fouyssac et al. 2022), which demonstrate the stimulatory effect of negative urgency on relapse to cocaine seeking, it can be hypothesized that during the induction of nicotine-seeking behaviour following abstinence, there may be an overlap between nicotine craving and instrumental deprivation (i.e., lack of the opportunity to press the levers). This interaction could have exacerbated the intensity of nicotine-seeking behaviour, potentially reducing the effectiveness of physical activity in modulating this behaviour. It will be important to investigate whether operant behaviour influences nicotine seeking.

Clinical studies on the effects of exercise on smoking cessation suggest that the intensity and timing of exercise during abstinence can impact its effectiveness (Everson et al. 2008; Klinsophon et al. 2017). Among temporarily abstinent smokers, moderate-intensity exercise has shown greater benefits for reducing craving than does vigorous-intensity exercise (Russell et al. 1988; Everson et al. 2008; Janse et al. 2009; Elibero et al. 2011), with the latter leading to increased tension, anxiety, or negative mood effects (Russell et al. 1988; Everson et al. 2008). Positive effects of exercise on craving or cessation were observed in 12-week programs(Marcus et al. 1999; Kunicki et al. 2022) but not in shorter 7-week programs (Ussher et al. 2003), with vigorous exercise sometimes providing only temporary relief from craving (Bock et al. 1999). Overall, these findings highlight the importance of considering the intensity and duration of exercise interventions in smoking cessation programs. In rodent studies, investigating the effects of wheel running in nicotine-deprived rats alongside heart rate monitoring devices could provide optimal exercise conditions for supporting nicotine cessation.

### The role of hippocampal neurogenesis in nicotine abstinence-induced depression

One hypothesis of depression involves reduced synthesis of new neurons in the hippocampus (Ernst et al. 2006; Snyder et al. 2011). In this context, our earlier study (Zaniewska et al. 2021) and the present investigation revealed that during protracted nicotine abstinence, rats developed a depression-like phenotype concomitant with a decrease in the number of immature neurons (DCX^+^) in the DG. Conversely, exercise (as shown in the present study), similar to the pharmacological agent lorcaserin (a 5-HT_2C_ receptor agonist) (Zaniewska et al. 2021), had an antidepressant effect on nicotine-deprived rats and increased the number of immature neurons in the DG. These findings suggest that depression-like symptoms in nicotine-deprived rats may stem from a decrease in the number of immature neurons expressing DCX. DCX, a microtubule-associated protein crucial for neuron migration and neocortical and hippocampal development, has been associated with significant developmental disorders in humans (lissencephaly) and mice (hippocampal lamination abnormalities), affecting learning and social behaviours (Corbo et al. 2002; Kappeler et al. 2007; Vukovic et al. 2013; Germain et al. 2013). Nevertheless, the impact of DCX expression in the DG on depression has yet to be explored, and its causal relationship with depression-like symptoms needs to be determined in future research.

### The role of hippocampal neurogenesis in nicotine-seeking behaviour

Currently, there are no data in the literature on the impact of wheel running on nicotine-seeking behaviour in relation to hippocampal neurogenesis. Studies on methamphetamine use have shown that intermittent access to the drug increases, while daily access reduces hippocampal neurogenesis in rats (Mandyam et al. 2008). Methamphetamine withdrawal increased hippocampal cell proliferation and survival (Recinto et al. 2012). In turn, exposure to wheel running– a neurogenesis-enhancing factor– during methamphetamine abstinence improved the extinction response and cue-induced reinstatement (Sobieraj et al. 2016). In the case of another psychostimulant, cocaine, limited access to the drug decreased cell proliferation, differentiation, and survival (Deschaux et al. 2014; Ahdoot-Levi et al. 2021), while extinction normalized cell survival (Deschaux et al. 2014). Increased cocaine administration decreased cell proliferation in the DG (Noonan et al. 2008), and withdrawal reversed these changes and improved neuronal maturity (Noonan et al. 2008). Reducing hippocampal neurogenesis enhanced context- or cue-induced cocaine seeking (Noonan et al. 2010; Deroche-Gamonet et al. 2019), while wheel-running exercise positively influenced cue- and drug-evoked cocaine seeking, although its effects on extinction responses are inconsistent (Zlebnik et al. 2010; Lynch et al. 2010; Peterson et al. 2014; Ogbonmwan et al. 2015; Zlebnik and Carroll 2015). Overall, it seems that the varying impact of exercise on seeking behaviour induced by stimuli associated with nicotine and other drugs of abuse may arise from the differing states of hippocampal neurogenesis during cessation of these substances. With regard to nicotine, more studies are required to assess neurogenesis levels during the induction of seeking behaviour.

In summary, animal studies have demonstrated that wheel-running exercise during nicotine abstinence can induce an antidepressant response in rats without affecting nicotine-seeking behaviour. The positive effects of running on mood during nicotine withdrawal may be due to a reduction in the loss of DCX^+^ neurons in the DG. This evidence supports the potential of physical activity as a non-pharmacological therapy for the treatment of nicotine addiction because it positively affects hippocampal neurogenesis changes caused by nicotine withdrawal.

Our study has certain limitations that should be addressed in future research. One key limitation is the duration of the observation period for developing neurons, as well as the variations in control groups, which may have affected the full capture of the observed effects. Notably, the study did not include a saline (SAL) group or a ‘yoked’ group, where nicotine (YNIC) would be administered whenever the paired rat self-administered nicotine (NIC). The inclusion of the YNIC group could have helped to distinguish the motivational aspects of nicotine intake from the pharmacological effects of nicotine itself. The inclusion of YNIC-LW/WR subgroups could provide additional insights into the effects of nicotine withdrawal on running behaviour, immobility in the FST, and neurogenesis in the NIC-WR group. Additionally, future studies are needed to determine whether the positive effects of running on depression-like behaviour in nicotine-deprived animals in the FST result from acute or long-term exposure to running. Moreover, given the growing evidence on sex differences in behavioural and molecular research, it will be crucial to assess whether our findings extend to females as well. Finally, the absence of mechanistic studies in our research limits our understanding of the role of neurogenesis in nicotine-related behaviours.

Further research is necessary to determine the optimal exercise parameters for effectively managing nicotine cessation.

## Electronic supplementary material

Below is the link to the electronic supplementary material.

Supplementary Material 1

## Data Availability

The datasets generated during and/or analysed during the current study are available from the corresponding author upon reasonable request.
