# Supplementary material for: The impact of voluntary wheel-running exercise on hippocampal neurogenesis and behaviours in response to nicotine cessation in rats
Source: Psychopharmacology (Berl). 2024 Oct 27;241(12):2585–607. doi: 10.1007/s00213-024-06705-7 (PMC11569017; doi:10.1007/s00213-024-06705-7)
Supplement: Supplementary file 1 — Supplementary Material 1 [file 213_2024_6705_MOESM1_ESM.pdf]

The impact of voluntary wheel-running exercise on hippocampal neurogenesis and behaviours in response to nicotine cessation in rats

Magdalena Zaniewska[1,2,3,*], Sabina Brygider[1,2], Iwona Majcher-Maślanka[1], Dawid Gawliński[2], Urszula Głowacka [1,†], Sława Glińska[4], Łucja Balcerzak[4]

[1]*Department of Pharmacology and Brain Biostructure, Maj Institute of Pharmacology, Polish Academy of Sciences, 12 Smętna Street, 31-343 Kraków, Poland;*

[2]*Department of Drug Addiction Pharmacology, Maj Institute of Pharmacology, Polish Academy of Sciences, Smętna 12 Street, 31-343, Kraków, Poland;*

[3]*Affective Cognitive Neuroscience Laboratory, Maj Institute of Pharmacology, Polish Academy of Sciences, 12 Smętna Street, 31-343 Kraków, Poland;*

[4]*Faculty of Biology and Environmental Protection, Laboratory of Microscopic Imaging and Specialized Biological Techniques, University of Lodz, Banacha 12/16, 90-237 Lodz, Poland*

[†]Current address: Department of Physiology, Jagiellonian University Medical College, 16 Grzegorzecka Street, 31-531 Kraków, Poland.

* Correspondence: zaniew@if-pan.krakow.pl; +48126623373 (M.Z.)

Supplementary Results

Voluntary wheel running does not alter lever presses during the various phases of the drug-seeking session

On abstinence Day 15, there were no significant differences in the number of active lever presses made under the FR schedule between rats with locked and unlocked running wheels (lever press × environment interaction ($F(1,20) = 0.93$, $p = 0.35$, $\eta^2_p = 0.044$), environment ($F(1,20) = 0.56$, $p = 0.46$, $\eta^2_p = 0.027$)). A significant main lever effect was observed ($F(1,20) = 20.17$, $p = 0.00022$, $\eta^2_p = 0.5$), indicating more active than inactive lever presses in both treatment groups ($p < 0.001$; Suppl. Fig. 1, left panel).

There were also no significant differences in the number of active lever presses during infusions between rats with locked and unlocked running wheels (lever press × environment interaction ($F(1,20) = 1.03$, $p = 0.32$, $\eta^2_p = 0.049$), environment ($F(1,20) = 2.31$, $p = 0.14$, $\eta^2_p = 0.10$)). A significant main lever effect was observed ($F(1,20) = 12.56$, $p = 0.002$, $\eta^2_p = 0.39$), indicating more active than inactive lever presses in both treatment groups ($p < 0.01$; Suppl. Fig. 1, middle panel).

There were also no significant differences in the number of active lever presses during time-out periods between rats with locked and unlocked running wheels (lever press × environment interaction ($F(1,20) = 0.38$, $p = 0.55$, $\eta^2_p = 0.018$), environment ($F(1,20) = 0.23$, $p = 0.64$, $\eta^2_p = 0.011$)). A significant main lever effect was observed ($F(1,20) = 9.38$, $p = 0.0061$, $\eta^2_p = 0.32$), indicating more active than inactive lever presses in both treatment groups ($p < 0.01$; Suppl. Fig. 1, right panel).

Supplementary Figures

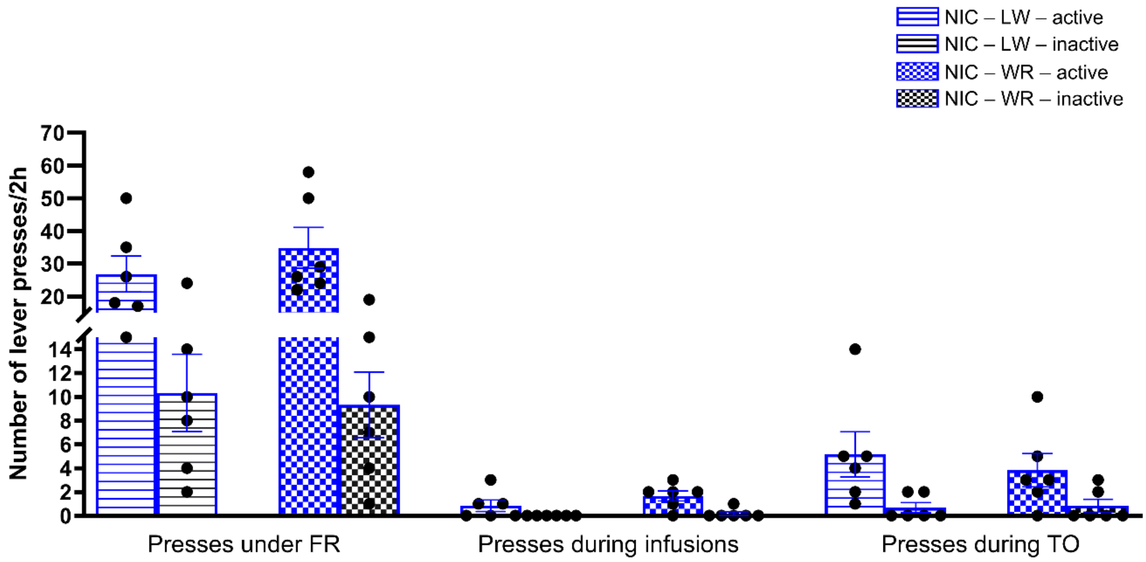

Suppl. Fig. 1 Effects of wheel-running exercise on nicotine-seeking behaviour. Rats were allowed to self-administer nicotine (NIC; 0.03 mg/kg/inf) in 2-h sessions. After 21 sessions, the rats entered a 14-day deprivation phase. On abstinence Day 1, the animals were transferred to cages equipped with either running wheels (WR) or locked wheels (LW) for the next 13 days of abstinence. The figure shows the effects of WR on the number of lever presses induced by NIC priming (0.4 mg/kg, sc) on abstinence Day 15 (n = 6 rats/group). Data are presented separately for lever presses made under the fixed ratio (FR) schedule, during infusions, and during the time-out (TO) period. These data correspond to Fig. 6i, which presents the total lever presses made during all phases of the drug-seeking session. The data are expressed as the means (±SEM). For lever presses under the FR schedule: $p < 0.001$: a significant effect of lever: (active vs. inactive). For lever presses during infusions or TO: $p < 0.01$: a significant effect of lever (active vs. inactive)
